# Supplementary material for: Cellular pathways during spawning induction in the starlet sea anemone Nematostella vectensis
Source: Sci Rep. 2021 Jul 29;11:15451. doi: 10.1038/s41598-021-95033-3 (PMC8322078; doi:10.1038/s41598-021-95033-3)
Supplement: Supplementary file 1 — Supplementary Legend. [file 41598_2021_95033_MOESM1_ESM.docx]

**List of Supplementary Figures and Tables:**

**Fig. S1:** KEGG metabolic map of fatty acid degradation.

**Fig. S2:** Real-time qPCR analysis before and during light or dark induction.

**Table S1:** Transcriptome analysis of all transcripts during spawning induction (Excel file).

**Table S2:** Enrichment of up- and down-regulated GO terms at 1, 2, 5 and 8 h during induction (Excel file).

**Table S3:** List of primers used in qPCR expression analysis.
