# Supplementary material for: Cellular pathways during spawning induction in the starlet sea anemone Nematostella vectensis
Source: Sci Rep. 2021 Jul 29;11:15451. doi: 10.1038/s41598-021-95033-3 (PMC8322078; doi:10.1038/s41598-021-95033-3)
Supplement: Supplementary file 4 — Supplementary Table S3. [file 41598_2021_95033_MOESM4_ESM.pdf]

Table S3: List of primers used in qPCR expression analysis.

Name	JGI ID	5'-Forward	3'-Reverse
Aqp	185753	495-GGCCATTGGACTGTGTGTCA	574-ATGATCGCGCAGGGTTGA
Clock	160110	87-GCATTGAAAGCATGGACGATAA	181-GAATCTATCCCGCCGTTTCTT
Cry	106211	952-CAACACCCGGAAGCTCTTTC	1031-CGCAACTGTCGCATTATTGC
Cry1a	168581	1129-GAGGGACTCAGGGCTTTTGAG	1248-AGGGCAGAACCATGGGATTT
Cry1b	106062	713-CTGCTTTGAAAAAGTGTGCTGAA	827-GGCGACAAACATCCGAATCT
FUE	244532	751-GAGATGATGATTCCTGCTAGCAAA	830-GCCCGTTCCTGTAAGTTCTTGA
Grin1	171792	1915-CTCGTCCTCGATCGACCTAAA	2006-GCCACCGTCGCGTAGATG
Helt	246249	118-GGCAAGAAAAAAGCAAATTCTGAA	207-CGCCAGCTCTGACAAGCA
TEF	39846	49-TGGGAAAGAAGATTGAAAAACAATG	137-TGAGCGCGCTTTGCTACA
Tuba1a	188665	183-CGTCCCCAGAGCAGTCTTTG	292-CCTCTTTGCCAGTGATCAGTTG
Wnt3	241352	380-GGCGTCGCGCATTCC	443-GATCACAGTCGCAGCTTTCTATTC

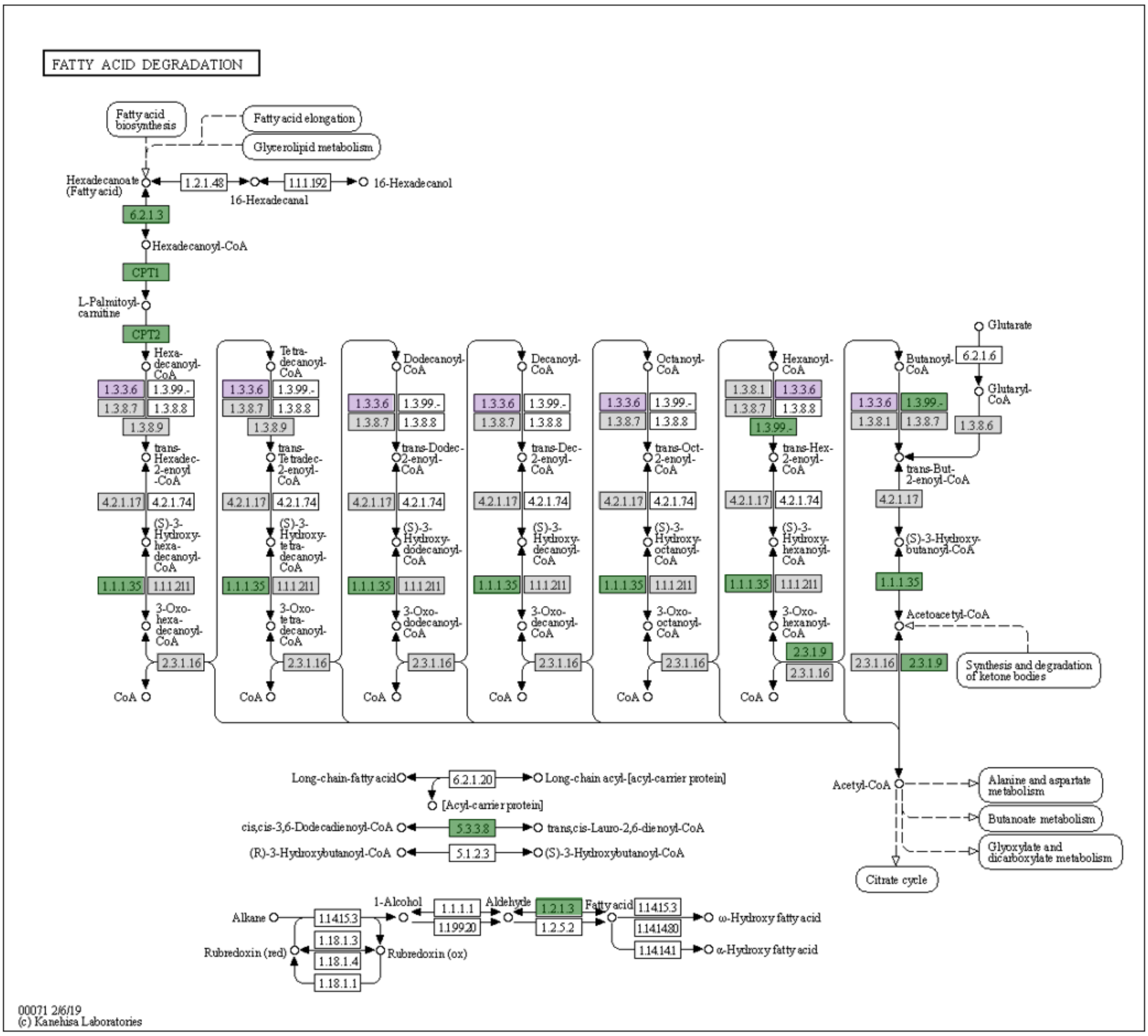

Fig. S1: KEGG metabolic map of fatty acid degradation. Map (nve00071) describes the breaking down of fatty acids to their metabolites, eventually generating acetyl-CoA. Green and purple boxes are significantly down- and upregulated transcripts, respectively. Gray boxes are transcripts that were identified in *Nematostella*. Numbers are KEGG identifiers.

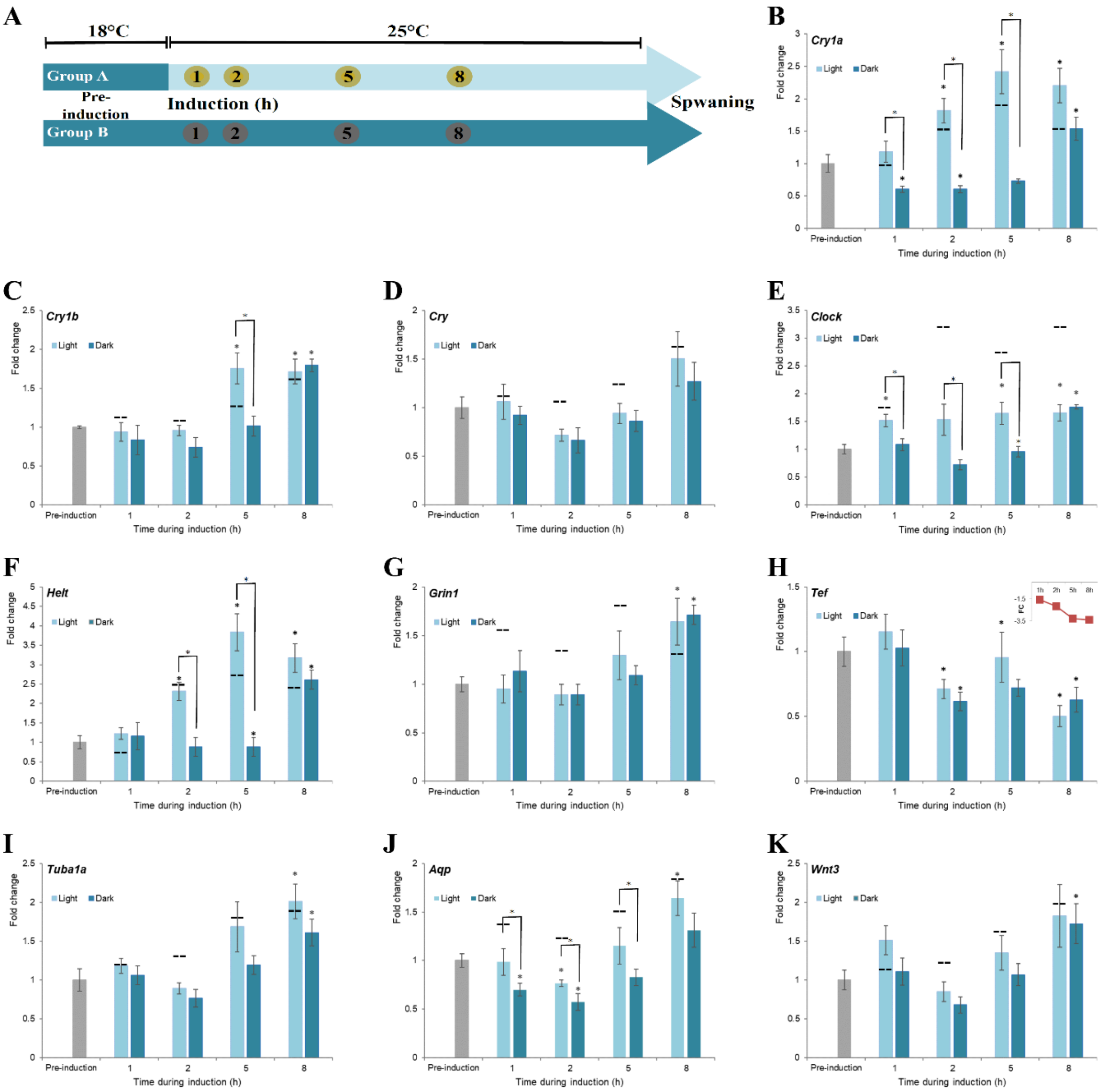

Fig. S2: Real-time qPCR analysis before and during light or dark induction. (A) Schematic representation of the qPCR experiment. Induction refers to time in hours from temperature stimulus to successful spawning. Temperature regime is indicated by black bars. Light regime for each experimental subgroup is indicated by light or dark arrow color. Sampling time points are indicated in circles. qPCR analysis of (B) *Cry1a* (168581), (C) *Cry1b* (106062), (D) *Cry* (106211), (E) *Clock* (160110), (F) *Helt* (246249), (G) *Grin1* (171792), (H) *Tef* (39846), (I) *Tuba1a* (188665), (J) *Aqp* (185753) and (K) *Wnt3* (241352). FUE (244532) was used as a normalizing gene. Results are presented as the mean fold change ± SE ($n \geq 4$). Asterisks indicate significant differences from the control ($p < 0.05$). Asterisks between bars indicate significant differences between light/dark treatments ($p < 0.05$). Black dashed lines mark the fold change of upregulated transcripts in the RNA-seq experiment and the small graph shows the RNA-seq experiment for the downregulated transcript *Tef*.
