# Supplementary material for: Cellular pathways during spawning induction in the starlet sea anemone Nematostella vectensis
Source: Sci Rep. 2021 Jul 29;11:15451. doi: 10.1038/s41598-021-95033-3 (PMC8322078; doi:10.1038/s41598-021-95033-3)
Supplement: Supplementary file 5 — Supplementary Figure S1. [file 41598_2021_95033_MOESM5_ESM.pdf]

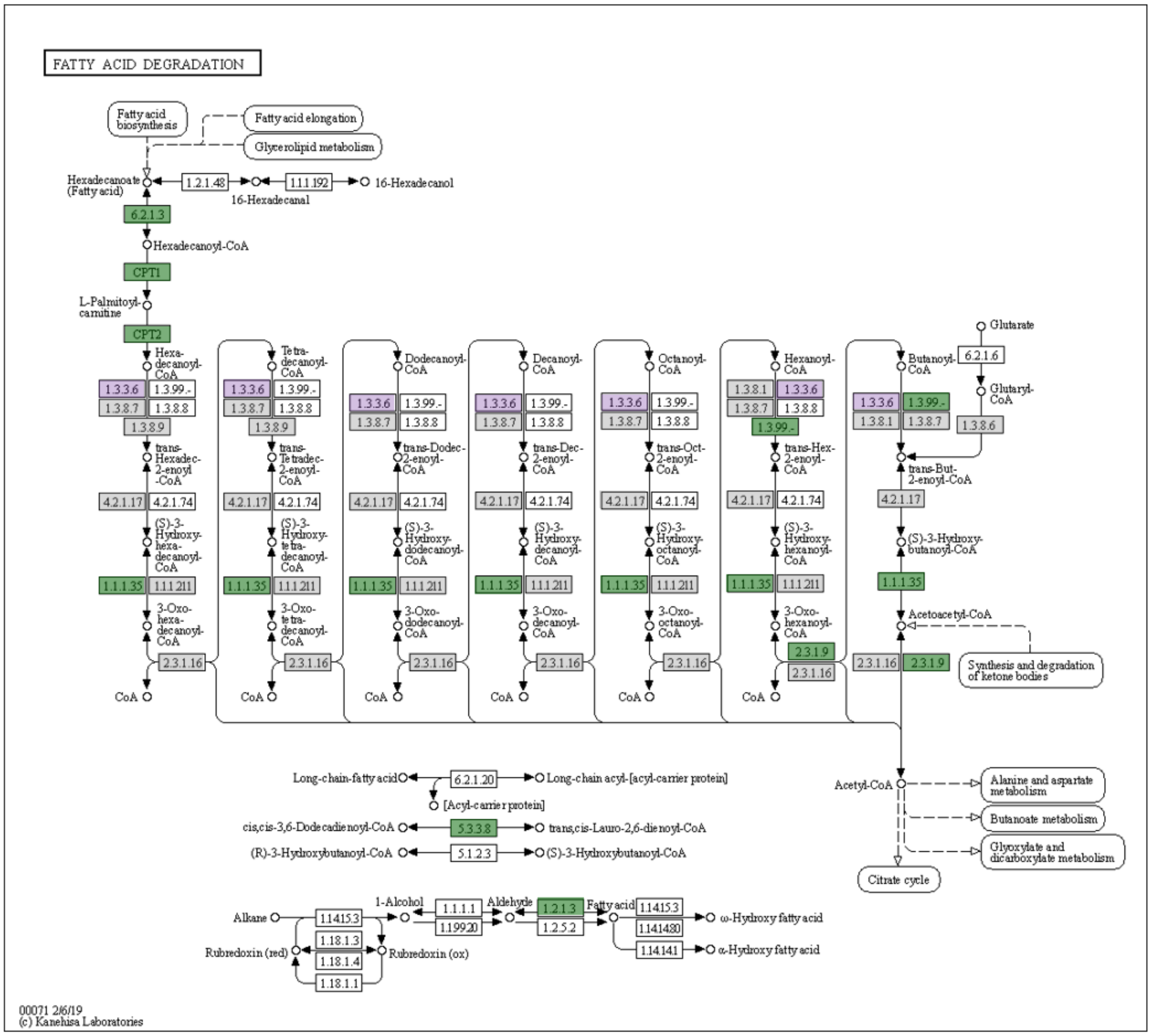

Fig. S1: KEGG metabolic map of fatty acid degradation. Map (nve00071) describes the breaking down of fatty acids to their metabolites, eventually generating acetyl-CoA. Green and purple boxes are significantly down- and upregulated transcripts, respectively. Gray boxes are transcripts that were identified in *Nematostella*. Numbers are KEGG identifiers.
