# Supplementary material for: Cellular pathways during spawning induction in the starlet sea anemone Nematostella vectensis
Source: Sci Rep. 2021 Jul 29;11:15451. doi: 10.1038/s41598-021-95033-3 (PMC8322078; doi:10.1038/s41598-021-95033-3)
Supplement: Supplementary file 6 — Supplementary Figure S2. [file 41598_2021_95033_MOESM6_ESM.pdf]

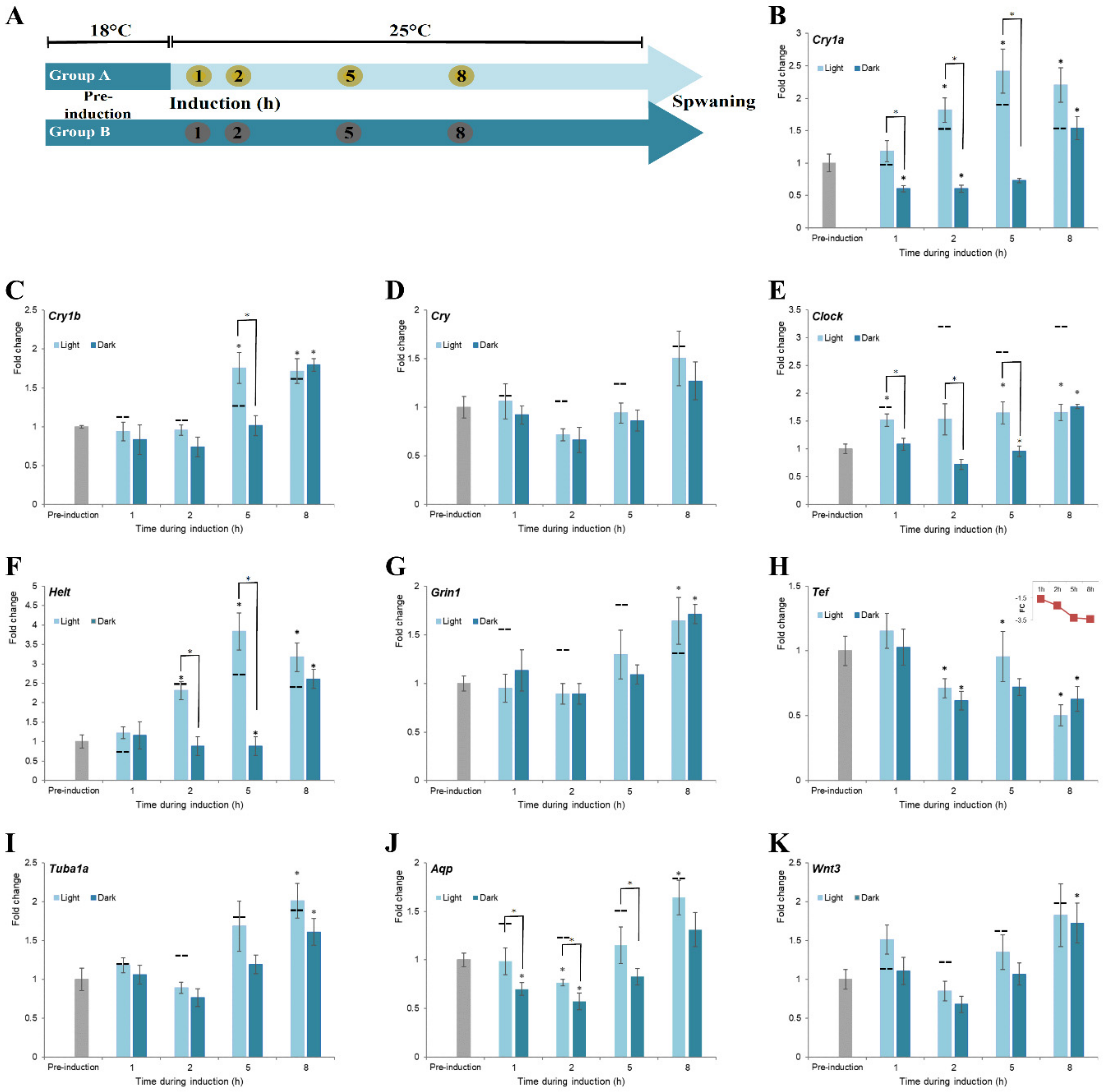

Fig. S2: Real-time qPCR analysis before and during light or dark induction. (A) Schematic representation of the qPCR experiment. Induction refers to time in hours from temperature stimulus to successful spawning. Temperature regime is indicated by black bars. Light regime for each experimental subgroup is indicated by light or dark arrow color. Sampling time points are indicated in circles. qPCR analysis of (B) *Cry1a* (168581), (C) *Cry1b* (106062), (D) *Cry* (106211), (E) *Clock* (160110), (F) *Helt* (246249), (G) *Grin1* (171792), (H) *Tef* (39846), (I) *Tuba1a* (188665), (J) *Aqp* (185753) and (K) *Wnt3* (241352). FUE (244532) was used as a normalizing gene. Results are presented as the mean fold change ± SE ($n \geq 4$). Asterisks indicate significant differences from the control ($p < 0.05$). Asterisks between bars indicate significant differences between light/dark treatments ($p < 0.05$). Black dashed lines mark the fold change of upregulated transcripts in the RNA-seq experiment and the small graph shows the RNA-seq experiment for the downregulated transcript *Tef*.
